# Potential Therapeutic Applications of Bee Venom on Skin Disease and Its Mechanisms: A Literature Review

**DOI:** 10.3390/toxins11070374

**Published:** 2019-06-27

**Authors:** Haejoong Kim, Soo-Yeon Park, Gihyun Lee

**Affiliations:** 1 College of Korean Medicine, Dongshin University, Naju-si, Jeollanam-do 58245, Korea; 2 Department of Ophthalmology, Otolaryngology & Dermatology, College of Korean Medicine, Dongshin University, Naju-si, Jeollanam-do 58245, Korea

**Keywords:** bee venom, alternative treatment, skin, cutaneous disease, mechanism

## Abstract

Skin is larger than any other organ in humans. Like other organs, various bacterial, viral, and inflammatory diseases, as well as cancer, affect the skin. Skin diseases like acne, atopic dermatitis, and psoriasis often reduce the quality of life seriously. Therefore, effective treatment of skin disorders is important despite them not being life-threatening. Conventional medicines for skin diseases include corticosteroids and antimicrobial drugs, which are effective in treating many inflammatory and infectious skin diseases; however, there are growing concerns about the side effects of these therapies, especially during long-term use in relapsing or intractable diseases. Hence, many researchers are trying to develop alternative treatments, especially from natural sources, to resolve these limitations. Bee venom (BV) is an attractive candidate because many experimental and clinical reports show that BV exhibits anti-inflammatory, anti-apoptotic, anti-fibrotic, antibacterial, antiviral, antifungal, and anticancer effects. Here, we review the therapeutic applications of BV in skin diseases, including acne, alopecia, atopic dermatitis, melanoma, morphea, photoaging, psoriasis, wounds, wrinkles, and vitiligo. Moreover, we explore the therapeutic mechanisms of BV in the treatment of skin diseases and killing effects of BV on skin disease-causing pathogens, including bacteria, fungi and viruses.

## 1. Introduction

Bee venom (BV), produced by honeybees (*Apis mellifera*), is one of the most well-known natural toxins. BV is a very diverse set of chemicals. It includes peptides such as melittin, apamine, adolapin, and MCD peptide, enzymes like phospholipase A2 (PLA2), hyaluronidase, acid phosphomonoesterase, and lysophosphofolipase, and it also contains various amines such as histamine, dopamine, and norepinephrine [1].

BV has long been used as a therapeutic substance. It generally has been administrated in the form of piercing directly with bee sting, or injecting extracted and purified BV with a syringe. In oriental medicine, BV is also injected into specific acupoints related with a disorder [2,3]. BV has been broadly used for reducing pain and suppressing inflammation in musculoskeletal disorders, such as osteoarthritis, rheumatoid arthritis, and lumbar pain [2,4,5,6], and in recent years, its therapeutic effects in treating neurological diseases like chronic neuralgia, Parkinson's disease, and amyotrophic lateral sclerosis have been reported [7,8]. Another recent study also showed that BV has a therapeutic effect on periodontal disease [9]. Accumulated evidence shows that BV has anti-inflammatory, anti-apoptotic, antifibrotic, and anti-atherosclerotic properties which support these therapeutic applications [10]. In addition, a number of recent studies have demonstrated antibacterial, antiviral, antifungal, and anticancer effects of BV [1,11,12,13,14,15,16,17].

**Figure 1:**
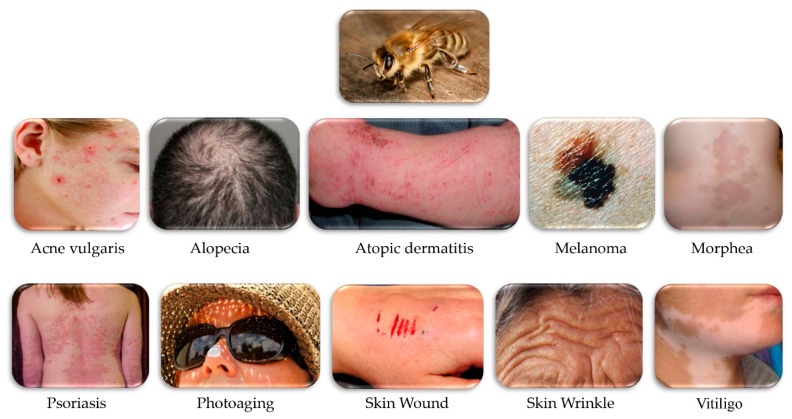
Skin diseases where the therapeutic application of bee venom (BV) has been studied.

Many reviews have highlighted the therapeutic value of BV, but none have focused on the effect of BV on skin diseases. To the best of our knowledge, this is the first review that summarizes the potential therapeutic mechanisms and applications of BV in skin diseases. They are shown ahead of discussion in order of clinical study, in vivo study, and in vitro study (Table 1, Table 2 and Table 3). To date, skin diseases where therapeutic application of BV has been studied include acne, alopecia, atopic dermatitis, melanoma, morphea, photoaging, psoriasis, wound, wrinkle, and vitiligo (Figure 1). The purpose of this review is to provide the present knowledge from a various experimental and clinical reports and to help researchers design a follow-up study from previous studies and diseases that are yet to be studied.

## 2. Therapeutic Effects of BV in Skin Diseases

### 2.1. Acne

Acne, marked by the development of papules, pustules, and nodules, is an inflammatory disorder which occurred on the sebaceous unit. Acne is generally observed on the skin of the face, breast, and back. Pathological features of acne include increased sebum secretion, inflammation, keratinization of sebaceous ducts, and bacterial colonization of sebaceous ducts [18,19]. Antibiotics, one of the various treatment options for acne, have been utilized to suppress inflammation by killing the causative bacteria [20]. However, frequent use of antibiotics poses the risk of side effects, like the appearance of resistant bacterial strains [21,22]. Therefore, there is a growing interest in acne treatments which have a higher therapeutic effect and fewer side effects [22,23]. There are currently many studies demonstrating that BV might be effective for acne vulgaris. 

#### 2.1.1. Clinical Studies

In a randomized double-blind control trial of Han et al., to examine the therapeutic effects of BV on acne, a total of 12 subjects received either skincare products containing BV or products without BV for 2 weeks. The BV group showed a notable advancement in the grading levels based upon the count of inflammatory and non-inflammatory lesions compared to the control. In the study, patients applying cosmetics containing BV showed a reduction by 57.5% in ATP levels, measured to assess a decrease in the count of skin microbes. These results show that cosmetics containing BV may be good candidates for therapeutic agents for acne [24].

In a prospective, non-comparative study of Han et al., 30 subjects with mild to moderate acne were recruited and managed with cosmetics containing BV twice daily for 6 weeks. All the volunteers showed significant improvement in the average visual acne grade compared to the start. The mean extent of improvement in acne grade after 6-weeks was 52.3%, and 77% of the subjects showed advancement in terms of whiteheads and blackheads, papules, pustules, and nodules 6 weeks later compared with the start of the treatment. There was no skin trouble noticed during the progress of the study. These results demonstrated that cosmetics containing BV showed a marked therapeutic effect on acne [25].

#### 2.1.2. In Vivo Studies

*Propionibacterium acnes* (*P. acnes*) is the main factor that induces inflammation in acne [26]. As a member of normal bacterial flora, *P. acnes* coexists in our skin, but its excessive proliferation plays a key role in the development of inflammatory acne. It contributes to the inflammatory response of acne by stimulating the production of inflammatory cytokines like IL-8, IL-1β and TNF-α from keratinocytes, sebocytes, and inflammatory cells [27]. 

An et al. intradermally injected *P.acnes* into the ears of mice, and then BV was applied to the right ear only to examine the therapeutic effects of BV on inflammatory skin disease induced by *P. acnes*. BV treatment significantly decreased the inflammatory cells infiltration, and the expression of TNF-α and IL-1β decreased significantly in the BV-treated ear as compared to the untreated ear. The expression of CD14 and TLR2 was also significantly inhibited by BV treatment in *P. acnes*-treated tissue. In addition, the transcriptional activity of NF-κB and AP-1 was noticeably inhibited after BV injection. These results indicate that BV may be beneficial in treating acne [28].

Melitin, the main component of BV, is cationic and is also a toxic peptide that causes hemolysis [29]. Interestingly, recent in vitro and in vivo studies have shown that these cytotoxic melittins can be used to treat inflammatory diseases by reducing excessive immune responses [30]. Lee et al. investigated the therapeutic efficacy of melitin as an alternative treatment for inflammatory skin diseases caused by *P. acnes*. In this study, melittin significantly decreased the swelling and granulomatous inflammation response, which were induced by intradermal injection of *P. acnes*, in the ear when compared to the ear that only *P. acnes* was injected into. The thickness of the ear injected with melittin showed a 1.3-fold decrease in comparison to the ears that only *P. acnes* was injected into. Moreover, melittin evidently downregulated the expression of TNF-α and IL-1β, which further led to remarkable suppression of CD14 and TLR2 expression. These outcomes indicate that the application of melittin has potential for the treatment of *P. acnes*-induced inflammatory skin disorder [31]. 

#### 2.1.3. In Vitro Studies

In vitro studies of Hari et al. using human keratinocytes and monocyte cells stimulated by heat-killed *P. acne* showed that BV reduced the production of IL-8, TNF-α, and IFN-γ in HaCaT and THP-1 cells. BV also suppressed TLR2 expression, which was induced by heat-killed *P. acnes*-induced, in HaCaT and THP-1 cells in a dose-dependent manner. The activation of TLR encourages the secretion of chemokines, pro-inflammatory cytokines, leukotrienes, and prostaglandins [32]. 

In the study of Han et al., BV decreased the production of IL-8 and TNF-α, which was caused by *P. acnes*, in THP-1 cell in a similar manner. In this study, BV showed low cytotoxicity against human keratinocytes and monocyte below 10 µg/mL [33]. These results indicate that BV might alternate antibiotic treatment for acne. 

Lee et al. tested melittin as a therapeutic agent in heat-killed *P. acnes*–treated keratinocytes. In this study, the injection of melittin considerably reduced the expression of diverse inflammatory cytokines, such as TNF-α, IL-8, IL-1β, and IFN-γ. Melittin treatment suppressed the expression of TNF-α and IL-1β via regulating NF-κB and MAPK pathways in keratinocytes. In this study, melittin did not influence the cell viability of HaCaT cells during 8 hours of treatment. [31]

### 2.2. Alopecia

Hair is regarded as one of the most crucial parts of a person’s look. Therefore, loss of hair could negatively affect self-worth and impair life quality. Genetic predisposition is the most common reason of hair loss, but stress is also believed to play a crucial role, specifically in the younger generation. Inflammation of the scalp is also associated with hair loss. According to a recent study, 74.1% of patients diagnosed with alopecia have inflammatory disorders, such as atopic and contact dermatitis. Mental illnesses account for 25.5% of the cases [34].

#### 2.2.1. In Vivo Studies

Park et al. investigated the preventive effect of BV on alopecia by application of BV or minoxidil (2%) to the dorsal surface of mice for 19 days. Dexamethasone (DEX) was used to induce catagen in mice. In this study, BV promoted hair growth in mice by decreasing the levels of 5α-reductase and increasing keratinocyte growth factor (KGF), which stimulates follicular proliferation [35]. 5α-reductase enzymatically catalyzed the conversion of testosterone into DHT that has a higher affinity against androgen receptors than testosterone, which led to stimulating hair loss by the expression of genes associated with hair follicle minimization [36]. Finasteride and dutasteride, which are currently used as hair loss treatment agents based on inhibiting 5α-reductase, can cause severe side effects, including sexual dysfunction, depression, and gynecomastia, which generally lead to treatment discontinuation [37,38]. In this study, no edema, erythema, irritation, and cytotoxicity were observed after BV treatment. These results suggest that BV may be used as a hair growth-promoting agent [35].

#### 2.2.2. In Vitro Studies

The length of hair relies on the duration of the anagen phase [39]. At any given time, hair, which is in anagen, catagen, and telogen phase, accounts for 90%, 1%, and 9% respectively [40]. In vitro studies using DEX-stimulated human dermal papilla cells (hDPCs) showed that BV elevates the proliferation of hDPCs and upregulates growth factors, including FGF7, FGF2, VEGF, and IGF-1 that keep hair in the anagen stage, and hence encourage hair growth in hDPCs [35]. BV presents the potential to be used as a treatment for hair loss, since it can stimulate hair growth by increasing hair growth factors and suppressing the progression to catagen phase. 

### 2.3. Atopic Dermatitis (AD)

Atopic dermatitis (AD) is a chronic and relapsing inflammatory skin disorder that is marked by a defective skin barrier, eczema, pruritus, dry skin, and an abnormal IgE-mediated allergic response to diverse external antigens [41].

The incidence of AD has increased considerably in recent years. About 1 to 3% of adults and a maximum of 20% of children have at some point suffered from AD [42]. Antihistamines, steroids, NSAIDs and immunosuppressants have been utilized to treat AD [43,44,45]. Regrettably, these medications have serious adverse effects, like nephrotoxicity and neurotoxicity [43,46]. Thus, natural substances have emerged as alternative therapeutic agents for immune disorders, such as AD because they are considered to have strong immunomodulatory effects and fewer side effects [47]. Emerging evidence indicates that BV alleviates AD via its anti-inflammatory mechanisms in clinical trials in vivo and in vitro studies.

#### 2.3.1. Clinical Studies

The disease management for AD is on the basis of hydrating the skin and restoring the collapsed epidermal barrier. Regular use of suitable moisturizer is an important part of therapy for AD because xerosis and barrier malfunction are the major symptoms of AD [48]. In the study of You et al., 136 subjects with diagnosed AD were randomly distributed in different groups and were made to apply either a moisturizer containing BV and silk protein or a moisturizer just without BV for 4 weeks. Subjects who applied emollient with BV showed significantly lower Eczema Area and Severity Index (EASI) and visual analogue scale (VAS) value compared to patients who applied emollient without BV. There were no outstanding differences in the incidence of side effects induced by BV on patients’ skin [49].

#### 2.3.2. In Vivo Studies

Gu et al. investigated the advancement of AD-like lesions caused by ovalbumin (OVA), which are major egg white proteins, and the mechanism of therapeutic action of BV simultaneously. Histological analysis of dorsal skin thickness indicated that intraperitoneal administration of BV reduced the symptoms of AD. BV inhibited inflammatory cytokines by decreasing IgE secretion, TNF-α, and thymic stromal lymphopoetin (TSLP). It also suppressed the infiltration of eosinophils and mast cells into the lesion. These outcomes indicate BV has a possibility to be developed as an alternative for AD treatment due to the effective inhibition of allergic skin inflammation in AD [50].

Kim et al. noted the relationship between hyperactivity of the complement system and the inflammatory response of AD. In a mouse model in which atopic lesions were induced by 1-chloro-2,4-dinitrobenzene (DNCB), subcutaneous injection of BV almost completely resolved the symptoms of AD. In this study, BV significantly increased the secretion of CD55, a complement formation inhibitor, from THP-1 cells, resulting in a significant reduction in serum C3C and MAC levels, which were evaluated as an indicator of complement system activation. These results suggest that BV may be able to manage AD by inducing CD55 production that inhibits activity of complement system [51].

Itching, a sensation that causes the desire to scratch, is the most outstanding symptom of AD, and continuous scratching further worsens AD symptoms [52]. In the compound 48/80-induced mouse skin scratching model used in study of Kim et al, intraperitoneal administration of BV mitigated scratching behavior in mice. This anti-scratching effect correlated with vascular permeability effects of BV. In this study, BV also significantly suppressed mast cell degranulation and the production of pro-inflammatory cytokines including TNF-α and IL-1β by downregulating the activation of the NF-κB pathway in compound 48/80-treated epidermal tissues. These outcome indicate that BV might be used to ameliorate compound 48/80-induced AD symptoms [47]. In traditional Oriental medicine, BV acupuncture (BVA), which involves injecting BV into acupoints, has been utilized to treat various chronic inflammatory disorder of humans. In a mouse model of thrombotic micro-angiopathy (TMA)-induced AD used in study of Sur et al., BVA treatment at BL40 acupoint in the ear significantly inhibited the expression of both Th1 and Th2 cytokines in lymph nodes and ear skin. The severity of ear skin infection symptoms as AD-like symptoms, including thickness, inflammation, and increased lymph node weight, were considerably soothed by BVA treatment. The proliferation and infiltration of T cells and the synthesis of IL-4 and IgE, which are typical Th2 allergic responses, were also suppressed by BVA treatment. Interestingly, BVA at BL40 acupoint showed a more pronounced inhibitory effect compared to the non-acupoint placed on the base of the tail. These outcomes suggest that injecting BV at specific acupoints successfully relieves AD-like lesions by suppressing allergic and inflammatory responses in a mouse with TMA-induced AD. [53]

Several studies have investigated the pharmacological effects of melittin, the main component of BV. In the study of An et al. using mouse with DNCB-induced AD, topical application of melittin significantly alleviated AD-like symptoms, such as dorsal skin thickness by decreasing the number of mast cell infiltration, CD4+ T cells and the serum level of IgE, IL-4, IFN-γ, and TSLP. In addition, whole BV and melittin restored the abnormal differentiation of epidermis by recovering the expression of filaggrin. These results indicate that melittin could be a suitable agent for the therapy of AD [54].

In the study of Kim et al., intraperitoneally-injected BV also improved OVA-induced AD-like symptoms, such as an increase in skin thickness, edema, erythema, and excoriation in mice by inhibiting mast cell infiltration, by decreasing filaggrin levels, and secretion of AD-related inflammatory chemokines and cytokines, including CD14, CD11b, IL-1β, TNF-α, TSLP, and an excessive IgE response. Taken together, these results confirm that melittin has therapeutic effects on AD-like symptoms. [55]

PLA2, another major component of BV, plays a central role in various cellular responses, such as signal transduction, and the regulation of inflammatory and immune responses and phospholipid metabolism [56,57]. In *Dermatophagoides farinae* extract (DFE)-induced AD mouse model used in the study of Jung et al., topical application of PLA2 significantly decreased the serum IgE, Th1, and Th2 cytokines. AD-induced histological changes and mast cells infiltration were alleviated by PLA2 application. Meanwhile, the depletion of regulatory T cells eliminated the anti-atopic effects of PLA2, which suggests that anti-atopic effects of PLA2 rely on the functions of regulatory T cells. Overall, the results demonstrated that topical application of PLA2 might ameliorate atopic skin inflammation [58]. 

#### 2.3.3. In Vitro Studies

The in-vitro study of An et al. using TNF-α/IFN-γ-treated human keratinocytes, melittin suppressed the production of chemokines, including CCL22 and CCL17, and pro-inflammatory cytokines such as IL-6, IL-1β, and IFN-γ by inhibiting the activation of NF-κB, STAT1, and STAT3 pathways. Modulating AD-associated cytokines and chemokines may therefore, offer therapeutic efficacy in patients with AD [54].

Filaggrin plays an important role in epidermal barrier function. The activation of STAT3 downregulates filaggrin and Th2-induced cytokines, including IL-4 and IL-13, which are known as activators of STAT3 signaling [59].

Kim et al. investigated anti-atopic effect of melittin using IL-4 and IL-13-stimulated human keratinocytes. In the study, melittin prevented filaggrin deficiency caused by IL-4/IL-13 and activation of STAT3 in keratinocytes. The report proposes that melittin may have a beneficial effect on the skin barrier function via inhibiting filaggrin deficiency by a reduction of IL-4, IL-13, pSTAT3, and TSLP expression [55].

### 2.4. Melanoma

Melanoma is a skin cancer which begins in melanocytes, which are cell types generally found in skin, eye and the bowel. The treatment for melanoma involves surgical removal, and adjuvant immuno-, chemo- and radiation therapy, which mainly destroy cancer cells by triggering apoptotic pathways [60].

#### 2.4.1. In Vivo Studies

Soman et al. investigated the anticancer effect of melittin on B16F10 mouse melanoma [61]. Melittin is a cytolytic peptide that inserts itself into lipid bilayer membranes followed by oligomerization to make pores on membrane [62]. Although this action can be used to destroy harmful cells, the nonspecific cytotoxicity, genotoxicity and hemolytic action of melittin have restricted its therapeutic usage [30]. To overcome this limitation, investigators used perfluorocarbon nanoparticles, synthetic nanoscale vehicles, which can deliver melittin to both, targeted tumors and premalignant lesions. After intravenous injection of melittin-loaded nanoparticles, tumor weight decreased significantly (~87% reduction). Melittin loaded on targeted nanoparticles induced cancer cell apoptosis via liberation of cytochrome c from mitochondria. In addition, histological analysis revealed a reduction in the number of proliferating cells, blood vessels and significant areas of necrosis. There were no apparent toxic effects in terms of changes in organ weight or serum chemical profile, and the levels of liver enzyme aspartate aminotransferase were significantly lower in the melittin group than the normal control [61].

#### 2.4.2. In Vitro Studies

BV caused the shift of intracellular Ca^2+^ concentrations in human melanoma A2058 cells. Changes in intracellular Ca^2+^ concentration generated reactive oxygen species (ROS) and collapsed mitochondrial membrane potential. As a result, apoptosis-inducing factor (AIF) and endonuclease G (EndoG) were noted to be translocated from mitochondria into the nucleus to carry out apoptosis. The inactivation of AKT and the activation of JNK were also observed in this process. Taken together, these experimental results supply a probable description for the potential mechanisms of BV in melanoma [60].

### 2.5. Morphea

Morphea is known as a local scleroderma and is a unique inflammatory disease that affects the skin and subcutaneous tissue, resulting in excessive accumulation of collagen, which eventually leads to fibrosis. Morphea is sometimes itchy but painless. It typically begins in red or purple skin areas and becomes thick and white [63].

The exact pathogenesis of morphea is unknown and the causes of morphea are generally considered as immune activation and inflammatory reaction, vascular endocardial damage, fibrosis, and nodularization. At the present time, there is no recommended medicinal treatment for morphea. Hwang et al, showed the successful outcome of BVA treatment in circumscribed morphea in a patient with systemic sclerosis [64].

#### Clinical Studies (Case Report)

A 64-year-old female presented with circular white areas (1 and 3 cm in diameter) and a heavy itch in the right lateral iliac crest. Subcutaneously, BVA was administered two times for the 1st week and once a week for the following 3 weeks along the edge of the superficial circumscribed lesions. After the first treatment, scores of sleep disturbance and itchiness dropped from 6 to 2 and from 8 to 4, respectively, on an 11 points numeric scale. At the 3rd visit, the patient reported that her itchiness had almost gone after two treatments, but it appeared intermittently. On the 5th visit, she reported that she did not feel the itchiness any more, and that she could sleep well. Her skin also improved. With a follow-up evaluation for 3 months, it was confirmed that her skin condition had improved drastically to resemble normal skin. Though there was a light itch at the site of BVA for around half a day following treatment, there were no other significant side effects during treatments. The result demonstrates the potential of BVA to be used as a local treatment for morphea [64]. 

### 2.6. Photoaging

Human skin is usually damaged by the exposure to ultraviolet (UV) ray of sunlight. Atmospheric ozone layer absorbs UVC, but both UVA and UVB arrive at the ground and have physiological effects [65]. In particular, UVB is regarded as one of the most hazardous environmental carcinogen because UVB irradiation can lead to the production of MMP-1 and MMP-3 in fibroblasts, inducing photoaging of the skin and progression of skin tumor [66].

#### In Vitro Studies

The in-vitro study of Han et al. using human dermal fibroblasts (HDF) irradiated by UVB, BV significantly decreased UV-induced MMP-1 and MMP-3 by 50–80% and 50–85%, respectively, compared to controls. It also reduced the expression of MMP-1 and MMP-3 mRNA. Moreover, BV promoted the recovery of the damage caused by UVB irradiation in HDF. One microgram/milliliter of BV exerted no remarkable effect on both cell viability and morphology. However, at the higher concentration of 10 µg/mL, BV treatment declined cell viability up to 90% [67]. These results hint that BV might be utilized as a potential protective agent for inhibiting photoaging. It is considered that BV allergy is mainly due to its allergic components, such as PLA2. Hyunkyoung et al. investigated the efficacy of PLA2-free bee venom (PBV) in preventing photoaging by comparing it with original BV. In this study, both BV and PBV decreased the levels of MMP-1and MMP-13 in HaCaT cells and MMP-1, -2 and -3 in HDF cells, which are induced by UVB, and restored the cell damage and production of collagen. In addition, both BV and PBV downregulated UVB-induced activation of ERK1/2 and p38 in HaCaT and HDF cells. However, the difference between the two is that BV shows a cytotoxic effect, whereas PBV showed some advantages in preventing skin wrinkle formation without significant cytotoxicity [68].

Activities of MMPs induced by UVB caused the degradation of collagen, which led to the loss of elasticity of skin ultimately forming wrinkles [69]. These results suggest that the application of PBV appears to be an effective method in preventing skin wrinkles and protecting the skin from exposure to UVB.

### 2.7. Psoriasis

Psoriasis is a chronic inflammatory skin disorder marked by well-circumscribed erythematous plaques covered with silvery white scales. The exact pathology is unknown and is believed to be related to the production of inflammatory cytokines and kemokinesis following the activation of T-cells and several types of white blood cells that rule cellular immunity [70]. Unfortunately, current therapies can only suppress psoriasis but not cure it. Studies are being planned to evaluate the efficacy of BV as a new therapy in patients with psoriasis.

#### Clinical Studies

In the study of Hegazi et al., patients received an intradermal injection of BV. Before treatment and after 3 months of therapy, Psoriasis Area and Severity Index (PASI) score and serum IL-1β were measured to evaluate the outcome of treatment. Both PASI score and serum levels of IL-1β showed a significant decrease upon BV treatment [71]. These results were in accordance with a study which reported a decrease in levels of IL-1β from 289.5 to 29.2 in patients with guttate psoriasis followed by improvement in psoriasis after tonsillectomy [72].

In this study, intradermal injection of BV showed a superior outcome to oral or topical propolis, which were used as positive controls. Interestingly, unlike most treatments used in psoriasis, no systemic adverse effects were observed in all subjects. This indicates that BV might be a safe new treatment option and could be utilized in patients who have renal or liver dysfunctions [71].

Recalcitrant localized plaque psoriasis (RLPP) is characterized by lesions counting under 10% of body surface area which does not respond to any topical and systemic treatment [73]. Eltaher et al. used BV as alternative curative agent for RLPP, and their results exhibited full recovery in 23 out of 25 (92%) of patients, whereas only one patient out of 25 (4%) showed a recovery in symptoms in the placebo group. PGA score and TNF-α levels were remarkably decreased in patients treated with BV compared to the group with placebo. The activity of inflammatory cytokines, including TNF-α, IL-6 and IL-1β, is considered to take responsibility for the development of psoriasis [74]; hence, decreasing their levels might contribute to improvement of the disease. In this study, no adverse effects were observed excluding erythema, mild swelling and slight pain at the spot of BV injection and these troubles eventually got better. Psoriatic lesions did not relapse following 6 months of observation. These results suggested that BV could be safe and effective management for RLPP [75].

### 2.8. Skin Wounds

Wound healing is the result of a complex and dynamic course of tissue repair that includes various cellular and molecular events [76]. Wound healing is achieved through five interrelated phases; hemostasis and formation of clot, fibroplasias and neovascularization, granulation tissue formation, re-epithelialization, and creation of new ECM and tissue remodeling [77,78]

#### In Vivo Studies

Han et al. examined the efficacy of BV on healing wound in mice. For research, full-thickness wounds were induced on the dorsal surface of mice, and mice were divided into BV control and Vaseline groups. All the treatments were applied on the gauze covering the wound. The expression of type 1 collagen showed an increase upon BV treatment compared to the other two groups. The speed of wound closure in the BV group was notably faster than in the other two groups. Histology also showed that BV induced remarkable progression of wound healing. In this study, BV reduced the level of fibronectin, TGF-β1, and VEGF but elevated type 1 collagen. The results demonstrated that BV promoted wound healing by inhibiting cytokines related with fibrosis, which led to a reduction of wound size and an increase in propagation of epithelium in mice with full-thickness excision. These results suggest that the topical application of BV could be highly useful in decreasing the sizes of wounds [79].

In diabetic patients, skin wound healing disorders are often the cause of morbidity and mortality. Insufficient recruitment of macrophages and neutrophils at the wound and damage to neovascularization are responsible for impaired diabetic wound healing [80,81].

Hozzein et al. examined the therapeutic effect and mechanism of BV on impaired wound healing caused by diabetes in a mouse model with type I diabetes mellitus. In this study, the rate of wound closure and recovery were increased in the BV-treated group in comparison to the control. Type I collagen showed significant restoration in diabetic mice treated with BV. In addition, the percentage of apoptotic macrophages decreased markedly in BV-treated groups compared to controls, which led to a significant increase of the phagocytic index. Furthermore, BV promoted angiogenesis via recovering Ang-1/Tie-2 signaling and increasing the expression of Nrf2, ERK, Akt/eNOS, and β-defensin-2, which shows a reduction in wound tissues in diabetic mice. These results indicate that BV could be applied as a new potential treatment for encouraging angiogenesis and repairing the impaired wound healing in diabetes [82].

### 2.9. Skin Wrinkling

Wrinkles are a change in appearance induced by ultraviolet rays, and the intrinsic aging process over a prolonged time. Both these factors induce collagen alteration, leading to skin aging. The desire to improve aging skin has led to the development of numerous cosmetics that slow down wrinkle formation. Currently, various ingredients have been added to theses cosmetics and BV is one of the added ingredients.

#### Clinical Studies

Han et al. assessed the beneficial effects of serum containing BV on facial wrinkles. The results of the application of BV-containing serum decreased average wrinkle depth, the total wrinkle area and count. Topical application of BV is considered to be safe for human skin since it showed no dermal irritation in animal researches [83]. Therefore, BV serum might be effective in improvement of skin wrinkles [84].

### 2.10. Vitiligo

Vitiligo is characterized by depigmentation of skin and hair. It is related to abnormal pigmentation resulting from melanocyte proliferation, melanogenesis, and migration or increases in dendricity [85,86]. Recently, phospholipase A2 of BV has been reported to stimulate melanocyte dendricity and pigmentation [86,87,88]. According to the authors, pigmentation which occurred around the injection sites and lasted a few months, had been observed after treatment with BV. One study investigated the effect of BV on the proliferation, melanogenesis, migration, dendricity, and signal transduction of human melanocytes.

#### In Vitro Studies

In the study of Jeon et al., BV treatment elevated the melanocyte proliferation around twice compared to the control in 1 week. By BV treatment, the expression of MITF-M protein increased to the maximum levels on day 3 and slowly decreased till day 5. BV also activated PKA, ERK, and PI3K/Akt signaling. Moreover, BV treatment increased the ratio of cells with more than two dendrites by 23% in a time-dependent manner. The results also showed that BV treatment led to a two-fold increase in the number of migrated cells as compared to the controls. BV-induced melanocyte dendricity and melanocyte migration showed complete inhibition upon pre-treatment with PLA2 inhibitor and aristolochic acid, and this suggests that BV-induced melanocyte dendricity and melanocyte migration occur via the activation of PLA2.

The results of the in-vitro study indicated that BV has a positive effect on melanocyte proliferation, melanogenesis, dendricity, and migration. The result suggest that BV has a potential for treatment of vitiligo by repigmentation in skin [89].

## 3. Inhibitory Effects of BV against Pathogenic Agent which is Related to Skin Disease

### 3.1. Bacteria

#### 3.1.1. *Propionibacterium Acnes*, Clindamycin-Resistant *P. acnes*, *Staphylococcus epidermidis*, and *Streptococcus pyogenes*

*Propionibacterium acnes*, *Staphylococcus epidermidis*, *Streptococcus pyrogenes*, and *Staphylococcus aureus* are microorganisms that originally exist on normal skin. During puberty, they proliferate rapidly and often contribute to the development of acne [90,91]. *P. acnes* infections mainly occur in the pilosebaceous unit. In contrast, aerobic organisms like *S. epidermidis*, *S. pyrogenes*, and *S. aureus* generally infect the sebaceous unit [92,93]. In the study of Han et al., BV shows bacteriostatic as well as bactericidal effects against *P. acnes*, clindamycin-resistant *P. acnes*, *S. epidermidis*, and *S. pyrogenes*. In this study, minimum inhibitory concentrations (MIC) of BV against *P. acnes*, clindamycin-resistant *P. acnes*, *S. epidermidis*, and *S. pyrogenes* were 0.086 μg/mL, 0.067 μg/mL, 0.104 μg/mL, and 0.121 μg/mL, respectively [33]. 

#### 3.1.2. *Staphylococcus aureus* and Methicillin-Resistant *Staphylococcus aureus (MRSA)*

*Staphylococcus aureus* is the main causative pathogen of Impetigo [94], Paronychia [95], and Staphylococcal-scalded skin syndrome [96]. BV exhibited a significant antibacterial effect against *S. aureus* in an in-vitro study using the disc diffusion method [11]. Although antibiotics effectively deal with *S. aureus* infections these days, the appearance of methicillin-resistant *S. aureus* (MRSA) is posing a challenge to global health systems at present [97]. 

In the study of Han et al. that investigated the antimicrobial effect of BV on MRSA strain in terms of minimum inhibitory concentrations (MIC) and minimum bactericidal concentrations (MBC), the MIC values of BV against MRSA CCARM 3366 and MRSA CCARM 3708 were 0.085 μg/mL and 0.11 μg/mL separately. Its MBC against MRSA 3366 and MRSA 3708 were 0.106 μg/mL and 0.14 μg/mL respectively. Interestingly, MRSA strains were more sensitive to the mix of BV and vancomycin or gentamicin than either ampicillin or penicillin alone. These outcomes showed that BV exhibits antimicrobial and enhancing antibacterial effects versus MRSA strains [13].

Choi et al. examined whether BV and melittin could suppress MRSA infections in vitro and in vivo. Surprisingly, BV showed outstanding antimicrobial effect in vitro, but strengthened the proliferation and infection of MRSA in vivo. All the mice, injected with MRSA USA300 and BV, died 18 h after infection, while only five died in the control 24 hours after infection. In addition, no outstanding difference was noticed in terms of the diameter of abscesses formed by MRSA USA300 even after BV treatment. 

Unlike BV, melittin showed remarkably better protection in the mice models of bacteremia and skin infection. Half of the mice survived over 24 h when 5mg/kg of melittin was injected 1 hour after bacterial infection and the abscess diameters were notably lower than the control [98].

### 3.2. Fungi

#### 3.2.1. Dermatophytes, *Trichophyton mentagrophytes*, and *Trichophyton rubrum*

*Trichophyton mentagrophytes* and *Trichophyton rubrum*, common dermatophytes, are known to cause various skin infections in humans and animals. Dermatophytes infect keratinized epithelium, the hair and nails. Tinea pedis is described as a dermatophyte infection on the soles of the feet and interdigital spaces; tinea cruris as an infection of the groin; tinea faciei as the facial infection; and tinea corporis as a fungal infection of the rest of the skin [99].

Yu et al. reported that BV showed significant antifungal effects against the two dermatophytes. In this study, 0.63 ppm of BV inhibited the growing of *T. menatgrophytes* by roughly 92% and *T. rubrum* by 26% after 1 h of incubation. Furthermore, BV exhibited much stronger antifungal activities than that of fluconazole, a standard antifungal agent utilized for the prevention and management of fungal infections. The results of this study suggest that BV could be developed as a natural antifungal drug [100].

Onychomycosis or tinea unguium which accounts for about half of all nail abnormalities is also caused due to dermatophytes, with the most common agent being *Trichophyton rubrum*. In the study of Park et al. that investigated the antifungal effects of BV, mellitin, apamin, and BV-based mists on *T. rubrum* indicated that BV and BV-based mist exhibited strong antifungal effect on *T. rubrum*. However, the isolated BV components, such as mellitin and apamin, showed no significant effect in hindering the growth of the fungal colonies [101].

#### 3.2.2. *Candida Albicans*

Cutaneous candidiasis is typically caused by *Candida albicans*, which exists as normal flora of human skin as well as in the gastrointestinal and genitourinary systems [102]. In the study of Lee et al., Sweet BV (SBV), which is made by removing enzymes and histamine known as allergens from BV, and BV exhibited an antifungal effect against 10 clinical isolates of *C. albicans* that were incubated from blood and the vagina.

In this study, SBV was noted to have much stronger antifungal activity than that of BV. The MIC values measured by the broth microdilution method diverge from 62.5 μg/mL to 125 μg/mL for BV, and in the case of SBV, from 15.63 μg/mL to 62.5 μg/mL. on the kill-curve assay, SBV acted similar to amphotericin B that was utilized as a positive control [103].

Park et al. demonstrated that melittin exerts its antifungal effect by inducing apoptosis. *C. albicans* treated with melittin exhibited an increase of ROS. In addition, markers that are indicators of apoptosis in yeast, involving externalization of phosphatidylserine, and fragmentation of DNA and nucleus fragmentation were observed. This study suggests that melittin exerts an antifungal effect by promoting apoptosis [104].

#### 3.2.3. *Malassezia furfur*

*Malassezia furfur*, a lipophilic yeast-like fungus exists as an opportunistic pathogen in human skin and causes disorders such as dandruff and pityriasis versicolor. 

In the study of Prakash et al., wherein 5 mg/mL BV was loaded onto the disc spread with *M. furfur*, a large zone of inhibition with an area of 86.9 mm^2^ was observed. Ketoconazole (200 mg/mL) was used as a standard reference and it showed an area of inhibition of 156.1 mm^2^. Since the doses of BV were in the range of 1–5 mg/mL, ketoconazole was applied at 5 mg/mL and it exhibited a suppression zone of 38.8 mm^2^.

In this study, BV showed good inhibition against *M. furfur*. Shampoos that can be bought in the commercial market are mostly made of chemicals and have many side effects. Therefore, supplements with natural compounds such as BV can be a better treatment for skin diseases caused by *M. furfur*. [105].

### 3.3. Viruses

#### Herpes Simplex Virus

Herpes simplex virus (HSV) invades skin and mucous membranes, harming keratinocytes and causing severe inflammation which is accompanied by small blister on the Erythema. Genital infections are mainly caused by HSV-2, while infections of other areas and the mouth are mostly caused by HSV-1 [106]. 

In the study of Uddin et al., a non-cytotoxic quantity of BV significantly suppressed the replication of HSV. These antiviral properties are mainly based on the virucidal activity of BV. Apart from antiviral activity, BV stimulated IFN-1, which could subsequently initiate antiviral signaling in the host cell and additionally suppress the replication of the virus. 

Uddin et al. also examined the antiviral effect of several components of BV to know which compounds in BV played a critical role in the virucidal effect of BV. Among those, only melittin in non-cytotoxic amounts exhibited similar effects to BV. Melittin directly destabilized the structure of virus particle, thereby suppressing viral infectivity. However, melittin was unable to interrupt the cell attachment and entry of the virus into the cells, and hence, once the cells were infected, it could not suppress viral infection and replication [12]. These results indicate that BV or melittin has the possibility to be a prophylactic or therapeutic agent in viral skin diseases.

## 4. Therapeutic Mechanisms of BV on Skin Diseases

Collective evidence from in-vitro experiments, in-vivo studies and clinical trials showed that BV has a potential therapeutic effect on skin diseases. The therapeutic mechanisms of BV mentioned above are as follows:

(1) In acne, TNF-α, IL-1β, TLR2, and CD14 expressions were remarkably decreased by BV treatment in *P. acnes*-injected tissues. In addition, the DNA-binding activity of NF-κB and AP-1 was noticeably inhibited by BV treatment [28]. BV reduced the expression of IL-8, TNF-α, and IFN-γ in HaCaT (keratinocyte) and THP-1 (monocytes) cells. It also suppressed TLR2 expression induced by heat-killed *P. acnes* in HaCaT and THP-1 cells [107]. Melittin significantly decreased TNF-α and IL-1β expression, leading to a noticeable suppression of TLR2 and CD14 expression in keratinocytes [31].

(2) In alopecia, BV promotes hair growth by reducing 5α-reductase expression and increasing KGF which stimulates follicular proliferation. BV increases the proliferation of hDPCs and upregulates growth factors, such as FGF7, FGF2, IGF-1, and VEGF, which maintain hair follicles in the anagen phase [35]. 

(3) In AD, BV suppressed the inflammatory cytokines by decreasing IgE, TNF-α, and TSLP levels. It also suppressed the infiltration of mast cells and eosinophils [50]. BV significantly increased the secretion of CD55, a complement formation inhibitor, from THP-1 cells, resulting in a significant reduction in serum C3C and MAC levels, which were evaluated as an indicator of complement system activation [51]. BV significantly inhibited mast cell degranulation and synthesis of pro-inflammatory cytokines, like TNF-α and IL-1β, by downregulating NF-κB activation [47]. The propagation and infiltration of T lymphocyte and the production of IL-4 and IgE, which are induced by Th2 type allergic responses, were suppressed by BV treatment [53].

Melittin decreased CD4+ T lymphocytes, mast cell infiltration and serum levels of IgE, IFN-γ, IL-4, and TSLP. Melittin also suppressed the production of chemokines, like CCL17 and CCL22, and pro-inflammatory cytokines involving IL-6, IL-1β, and IFN-γ by inhibiting the activation of NF-κB, STAT1, and STAT3 signaling pathways in keratinocytes [54]. Moreover, melittin inhibited filaggrin deficiency induced by IL-4 and IL-13 in keratinocytes. In addition, it suppressed mast cell infiltration and AD-related inflammatory molecules, such as CD14, CD11b, IL-1β, TNF-α, and TSLP and exaggerated IgE response [55]. The application of PLA2 significantly suppressed the increase in serum IgE and Th1 and Th2 cytokines. It also attenuated the infiltration of mast cells and histological changes [58]. 

(4) In melanoma, BV induced fluctuation in intracellular Ca^2+^ concentrations which increased the levels of ROS and collapse of membrane potential of mitochondria. As a result, AIF and EndoG are translocated from mitochondria into the nucleus to initiate apoptosis [60]. Melittin, loaded on targeted nanoparticles, induced apoptosis of cancer cell via release of cytochrome c from mitochondria [61]. 

(5) In morphea, the therapeutic mechanism of BV has not been studied yet. 

(6) In photoaging, BV significantly decreased UVB-induced MMP-1 and MMP-3 expression in HDFs [67]. BV also decreased the levels of MMP-1 and MMP-13 in HaCaT cells, and MMP-1, -2, and -3 in HDF cells, which are induced by UVB. Moreover, cell damage and production of collagen were restored by BV treatment. Furthermore, BV treatment downregulated UVB0-induced activation of p38 and ERK1/2 in HaCaT and HDF cells [68]. 

(7) In psoriasis, a notable reduction in the serum level of IL-1β was observed upon BV treatment [71]. 

(8) In skin wound healing, BV treatment elevated type 1 collagen expression and decreased the levels of TGF-β1, VEGF, and fibronectin, which are cytokines associated with fibrosis [79]. In diabetic wound healing, BV treatment markedly restored type 1 collagen expression. BV significantly decreased the percentage of apoptotic macrophages. In addition, BV promotes angiogenesis via recovering Ang-1/Tie-2 signaling and increasing the expression of Nrf2, ERK, Akt/eNOS and β-defensin-2, which are normally downregulated in wounded tissues [82]. 

(9) In wrinkled skin, the therapeutic mechanism of BV action is yet to be explored. 

(10) In vitiligo, BV significantly increased melanocyte proliferation, melanogenesis, dendricity, and migration. Hence, BV activated PKA, ERK, and PI3K/Akt signaling and increased MITF-M protein expression [89].

## 5. Discussion

Although no severe adverse effects were accounted from the studies reviewed here (Table 4), it cannot be ruled out that BV might cause fatal adverse reactions such as anaphylaxis [108]. Thus, physicians who use BV should be careful when administering BV to patients. In clinics, a skin test is used to determine whether BV treatment is suitable for individual patients, however, negative results of a skin test do not always guarantee safety [109]. Furthermore, one case report showed that anaphylaxis may occur in patients who have had no adverse reaction after former BV therapy [110]. Since anaphylaxis can occur under any circumstances, an emergency kit in accordance with the guidelines for management of anaphylaxis should always be kept ready. Meanwhile, one retrospective case study reported that the mean time to onset of anaphylaxis after BV therapy was 21.75 min [111], therefore, it is necessary to monitor the patient for at least 30 min after BV treatment. One recent study reported that high levels of basal serum tryptase increased the risk of severe anaxphylaxis [110]. As per this information, even if an injection of BV did not cause anaphylaxis in the past, if the basal serum tryptase is elevated at a certain time for some reason, anaphylaxis may occur when BV is injected. If this hypothesis is correct, the specific physiological state of the body at the point of BV injection may be a strong risk factor for the development of anaphylaxis. The analysis of safety of BV treatment will be a crucial factor in determining the value of BV as a therapeutic agent. We hope that further studies on prediction factors to prevent anaphylaxis upon BV administration will be conducted.

In this review, we surveyed the reports that showed the cytocidal effect of BV on pathogenic microorganisms that cause skin diseases as well as have a therapeutic effect on skin diseases. BV showed a significant inhibitory effect against various bacteria, fungi and viruses, and these results show potential applications of BV for diseases wherein the microbial agent is the main therapeutic agent. We expect further studies that examine the effect of BV on the treatment of various skin infections.

Treatment of warts by subcutaneous injection of BV is already being practiced in oriental medicine clinics in Korea. So, we believe that there would be a study that shows the therapeutic effects of BV on warts, but there have been no documented reports were warts were treated by using BV. Warts is known to be caused by skin infection with human papilloma virus (HPV). BV may also have a virucidal effect on the HPV virus that causes warts, as it is reported to have antiviral properties [12]. We look forward to further research about using BV in the management of warts. Furthermore, we hope that clinicians who use BV for the treatment of skin disorder actively report their cases. 

Treatment with natural substances is expected to have fewer side effects than with conventional medicine, but this comparison should ensure sufficient therapeutic effects. Many studies reviewed here have shown the ability of BV to reduce inflammatory cytokines and the disease-causing microbes; however, the status of BV among the current treatments is not very clear. Using current commercial drugs as positive controls in the future studies will help assess the precise therapeutic effect of BV. However, it may not be accurate to conclude that BV is meaningless as an alternative treatment because conventional medicine shows a greater magnitude of change in vitro studies. Because life has a very complex and organic structure, reactions to certain substances can be different between at the cell level and at the living level. Therefore, in order to ultimately determine whether BV has a therapeutic effect or not, it is necessary to evaluate how much change is made by BV in the lesion in the animal or human, not just in the cell. In addition, even if the efficacy of BV is lower than conventional therapy, it can be valuable as a therapeutic agent if it can make enough improvement of disease.Of course, in-vitro study can easily help in the analysis of molecular mechanisms and it plays an important role in providing hypotheses for follow-up research at a low cost; however, clinical trial and in-vivo study are necessary to decide the dosage and appropriate use of BV. Meanwhile, such studies are also very important in identifying the side effects of BV. Choi et al. conducted in vitro and in vivo studies to examine whether BV and melittin are able to suppress MRSA infections. Surprisingly, BV showed outstanding antimicrobial activity in vitro, but strengthened the proliferation and infection of MRSA in vivo [98]. Among 25 studies on 10 diseases surveyed this time, 15 studies were on acne and AD. The number of studies on the other eight diseases was not sufficient to conclusively assert the therapeutic role of BV. Especially, for vitiligo and photoaging, only in vitro studies have been carried out. We look forward to additional studies in the form of a clinical trials and more in vivo studies for the remaining eight diseases.

In this review, we have tried to investigate not only the therapeutic effects of BV, but also its acting mechanism. In the case of much studied acne and AD, there is considerable information about the mechanism of BV action. However, despite a limited number of studies, therapeutic mechanisms of BV action in alopecia, melanoma, photoaging, wound healing, and vitiligo were also found. However, no studies have been carried out for morphea, psoriasis and skin wrinkles. Despite many studies, the precise use of BV in treatment has not been accurately identified. We look forward to further studies that examine the molecular mechanism of BV treatment.

This review only dealt with melanoma in relation to skin cancer, but there was also a study that tested the efficacy of melittin in relation to the treatment of squamous cell carcinoma (SCC). SCC has the second highest prevalence of skin cancer after melanoma [112], with 700,000 new cases occurring each year [113]. The major risk factors of SCC are ultraviolet light and ultraviolet light absorbed by skin cells' DNA, including keratinocyte, causing genetic and epigenetic changes in these cells. In particular, studies have shown that p53 and the RAS pathway are responsible for this malignant transformation [114]. Do et al. demonstrated that the combination of melittin and 5-FU, which is used as topical treatment for SCC, increased the cancer-killing effect and reduced the cytotoxicity on normal keratinocyte [115]. Despite delicate data collection, there were studies that were missed. In the data collection process for preparing a review paper, a search method that can scan not only the title of the paper but also the contents of the paper should be considered.

It is not necessary to use only one method to treat diseases nor is it needed to replace the conventional drugs completely with natural substances. We expect that there is potential that a combination of BV and conventional medicine could prove to be a valuable therapeutic asset and could minimize adverse effects. We look forward to various types of follow-up research using BV.

## Figures and Tables

**Table 1:** Clinical study on therapeutic application of bee venom for skin disease.

Disease	Model	Venom/Compound/(Bee Species)	Dose (Administration Method)	Results	Mechanism/Molecular Response	Reference
**Acne**	Human DB, RCT (n = 12)	Cosmetic containing BV (*Apis melifera*)	0.06 mg/mL, Cosmetic 4 mL twice daily for 2 weeks (Applied to whole face)	Significant improvement of KAGS score (*p* < 0.01), 57.5% decrease of ATP level which indicate MO level (*p* < 0.01)	Not reported	[24]
**Acne**	Human (n = 30)	Serum containing BV (*Apis melifera*)	Not reported, Serum 0.7–0.9 g twice daily for 6 weeks (Applied to whole face)	Significant improvement (52.3%) of MCAGS score after 6 weeks (*p* < 0.001) Open and closed comedones were significantly decreased (*p* < 0.001). Significant decrease in papules (*p* < 0.05)	Not reported	[25]
**Atopic dermatitis**	Human DB, RCT (n = 114)	Emollient containing BV (*Apis melifera*)	Not reported, twice daily for 4 weeks (Applied to entire body)	Remarkable reduction of EASI score in comparison to control (*p* < 0.05). VAS score for pruritus was notably declined compared with control (*p* < 0.05). TEWL value were not notably different between two groups.	Not reported	[49]
**Psoriasis**	Human RLPP patients DB, RCT (n = 50)	BV (*Apis melifera*)	0.05 mL/cm^2^ (intradermal injection around psoriatic lesion)	BV treatment group showed significant lower PGA scores against placebo group (*p* < 0.001). During the follow-up period of 6 months, psoriasis did not recur.	TNF-α was notably decreased compared to control (*p* < 0.05).	[75]
**Psoriasis**	Human patients with localized plaque psoriasis (n = 48)	BV (*Apis melifera*)	Starting with 0.01 µL, increasing 0.01 µL every injection untill arriving 1 µL (Intradermal, twice weekly) TP: topical propolis twice daily OP: oral propolis 1 g/day by capsule	PASI score was significantly decreased decreased after treatment (*p* < 0.01). Much more reduction than TP and OP. The highest reduction in (TP + OP + BV) group.	Serum IL-1β was significantly decreased after treatment (*p* < 0.05). Much more decrease than TP and OP.The highest decrease in (TP + OP + BV) group.	[71]
**Scleroderma**	A case report: 64-year-old Korean woman, White circular lesion on the right lateral iliac crest	BV (*Apis melifera*)	Dried BV 1 g dissolved in 10000cc water. Total volume under 0.2 mL. twice weekly (subcutaneous, along the margins of the lesion)	On a 11-point numeric scale (NRS 11), average score of itch declined from 8 to 4 and sleep disturbance from 6 to 2, respectively. On the fifth visit, patient stated that she no longer felt an itch and had no sleep disturbance due to itching. Three months later, the follow-up evaluation showed that the condition of the skin was close to normal skin.	Not reported	[64]
**Wrinkle**	Human, Double blind (n = 22)	Serum containing BV (*Apis melifera*)	BV 0.006% serum 4 mL twice daily for 12 weeks (Applied to whole face)	The average visual grade (SKWGS) of all patients with BV serum significantly improved (11.83% decrement) (*p* < 0.001). Total area, count and average depth of wrinkle were significantly decreased (*p* < 0.05).	Not reported	[84]

Abbreviations: ATP: Adenosine triphosphate, DB: double-blind, EASI: eczema area and severity index, KAGS: Korean Acne Grading System, MCAGS: Modified Cook’s Acne Grading Scale, MO: micro-organism, PASI: psoriasis area and severity index, PASI: psoriasis area and severity index, PGA: physician global Assessment, RCT: randomized controlled trial, RLPP: recalcitrant localized plaque psoriasis, SKWGS: south Korean wrinkle-grading system, TEWL: transepidermal water loss, TNF-α: tumor necrosis factor-α, VAS: visual analog scale.

**Table 2:** In vivo studies on therapeutic application of bee venom for skin disease.

Disease	Model	Venom/Compound/(Bee Species)/	Dose (Administration Method)/Control	Results	Mechanism/Molecular Response	Reference
**Acne**	8-week ICR mice, P. acnes intradermally injected into both ears. (n = 30)	BV (*Apis melifera*)	1 µg blended with 0.05 g Vaseline (topical, on the right ear) NC: P.acnes only PC: vaseline applied to left ear	Ear thickness was reduced three-fold after 24 h compared to NC (*p* < 0.05). Swelling, erythema and inflammatory reactions were reduced.	TLR2 and CD14 expression is significantly inhibited. DNA-binding activity of NF-κB and AP-1 is remarkably inhibited compared to NC and PC (*p* < 0.05). Inhibiting the NF- κB signaling pathways.	[28]
**Acne**	8-week ICR mice, P. acnes intradermally injected into both ear. (n = 30)	Melittin (*Apis melifera*)	100 µg blended with 0.05 g Vaseline (topical, on the right ear) NC: P.acnes only PC: vaseline applied to left ear	Ear thickness was reduced 1.3-fold after 24 h compared with NC (*p* < 0.05). Swelling and granulomatous response were markedly reduced.	Significant reduction of TNF-α, IL-1β, IL-8, IFN-γ compared with NC and PC (*p* < 0.05). DNA-binding activity of NF-κB and AP-1 is remarkably inhibited compared to NC and PC (*p* < 0.05). Melittin significantly reduced the phosphorylation of IKK, IκB and NF- κB. Inhibiting the NF- κB and MAPK signaling pathways.	[31]
**Alopecia**	6-week female C57BL/6 mice, catagen phase induced on dorsal skin by dexamethasone.	BV (*Apis melifera*)	Three CONC: 0.001% 0.005% 0.01% 100 µL each Once daily for 19 day (Applied to dorsal skin) NC:dexamethasone only PC: minoxidil 2% 100 µL	Hair growth promoted notably in a dose-dependent manner at all doses. 0.01% BV resulted in the greatest increase in hair growth compared to PC (*p* < 0.05).	KGF expression is significantly increased compared with NC (*p* < 0.05). 5*α*-reductase significantly decreased compared with NC (*p* < 0.05).	[35]
**Atopic dermatitis**	DNCB induced atopic dermatitis in 7-week male Balb/c mice (n = 8)	BV (*Apis melifera*)	0.3 mg/kg (subcutaneous) PBS	Dryness, hemorrhage, excoriation, edema and redness were almost completely restored.	Serum C3C and MAC were significantly decreased after BV injection compared to PBS injection (*p* < 0.001). Serum-secreted CD55 were significantly elevated compared with PBS injection (*p* < 0.001). BV increased CD55 production in THP-1 cells	[51]
**Atopic dermatitis**	OVA-induced atopic dermatitis in 6-week female Balb/c mice (n = 25)	BV (*Apis melifera*)	Three doses: 1 µg/Kg, 10 µg/Kg, 100 µg/Kg twice a week for 2 weeks (intraperitoneal) NC: untreated PC: OVA only	Bleeding, erythema, eczema, and dryness were significantly reduced. Dorsal skin thickness was remarkably reduced in a dose-dependent manner compared to PC (*p* < 0.05), the greatest decrease in BV 100 group.	Significant reduction of mast cell infiltration in BV 10 and 100 group compared with PC (*p* < 0.05). Serum IgE levels were reduced, the greatest decrease in BV 100 group. Significant reduction of TNF-α in BV 10 and 100 and TSLP in BV 100 group compared with PC (*p* < 0.05).	[50]
**Atopic dermatitis**	DNCB induced atopic dermatitis in 6-week female Balb/c mice (n = 45)	Melittin (*Apis melifera*)	Three doses: 100 µg, 200 µg, 500 µg blended with placebo (topical, to dorsal skin) Placebo only	Dorsal skin thickness was notably decreased in comparison to placebo group (*p* < 0.05)	Mast cell infiltration was significantly decreased compared with control (*p* < 0.05). Serum IFN-γ, IL-4, IgE and TSLP were markedly decreased in melittin 200 and 500 group compared to placebo group (*p* < 0.05). CD4^+^ and CD3^+^ were significantly decreased in melittin 500 (*p* < 0.05).	[54]
**Atopic dermatitis**	Chicken OVA-induced atopic dermatitis in 6-week female Balb/c mice (n = 25)	Melittin (*Apis melifera*)	Three CONC: 1 µg/Kg, 10 µg/Kg, 100 µg/Kg (intraperitoneal) NC: untreated PC: OVA only	Dorsal skin thickness was significantly reduced in comparison to PC (*p* < 0.05), the greatest decrease in BV 100 group. Edema, erythema and excoriation were improved in melittin group.	Melittin significantly improved OVA-induced filaggrin deficiency (*p* < 0.05). CD14 and CD11b were significantly decreased in melittin 100 group compared to PC (*p* < 0.05). Mast cell infiltration was remarkably decreased in melittin 10 and 100 group compared to PC (*p* < 0.05). Serum IL-1β, TNF-α was notably decreased in all dose compared to PC (*p* < 0.05). Serum TSLP was remarkably decreased in melittin 100 compared to PC (*p* < 0.05). Skin IL-13 mRNA was significantly declined in melittin 100 compared with PC (*p* < 0.05).	[55]
**Atopic dermatitis**	DFE/DNCB-induced atopic dermatitis in 7–8-week female Balb/c mice (n = 25)	PLA2 (*Apis melifera*)	Two doses: 16 ng/ear, 80 ng/ear (Applied to ear skin) NC: DFE/DNCB only PC :dexamethasone 50 µg /ear	Ear thickness was notably decreased in all doses compared to NC (*p* < 0.001), not more than PC. AD-like skin lesions were significantly suppressed by PLA2.	Th1 cytokines (TNF- α, IL-6 and IFN-) and Th2 cytokines (IL-4 and IL-13) were remarkably decreased in comparison to NC (*p* < 0.05), no more effective than PC. Epidermal hyperplasia and lymphocyte infiltration were significantly attenuated by PLA2 in a dose-dependent manner compared with control (*p* < 0.01–*p* < 0.05), no more effective than PC. PLA2 has the potential to counteract AD-like skin lesion-associated inflammation responses via the induction of Tregs.	[58]
**Atopic dermatitis**	Compound 48/80-induced atopic dermatitis in 6-week Balb/c mice (n = 32).	BV (*Apis melifera*)	Two doses: 0.01 mg/Kg 0.1 mg/Kg (intraperitoneal) PC: Compound 48/80 only	Scratching behavior caused by compound 48/80 was decreased by 75% and 87% compared with PC in BV 0.01 and 0.1 respectively. (*p* < 0.05) Vascular permeability of the skin was decreased by 33.3% and 70.7% compared with PC in BV 0.01 and 0.1 respectively. (*p* < 0.05)	Mast cell degranulation was remarkably decreased in a dose-dependent manner compared to PC (*p* < 0.05). TNF-α and IL-1β were significantly suppressed in skin tissue by BV treatment. BV inhibited activation of NF- κB, which was induced by compound 48/80.	[47]
**Atopic dermatitis**	Trimellitic anhydride -induced atopic dermatitis on ear skin in 10-week male Balb/c mice (n = 50).	BV (*Apis melifera*)	0.3 mg/Kg, Once daily for 14 day (subcutaneous, acupuncture bilateral point BL40) NC: TMA treated PC: prednisone BVNA: BV at non acupoint; base of tail	BV at BL40 acupoint significantly relieved the AD symptoms. Thickness of ear and weight of lymph node were remarkably decreased compared to NC (*p* < 0.001). All results not better than PC but similar to BVNA indicated no healing effect on AD-like symptoms.	Serum IL-4 and IgE was notably declined compared to NC (*p* < 0.001). Number of CD4 and CD8 positive cells was notably declined in comparison to NC (*p* < 0.01). TNF-α, IFN-γ, IL-2, IL-4, IL-10 and IL-12 concentration in auricular lymph node were remarkably decreased compared to NC (*p* < 0.001–*p* < 0.05).	[53]
**Melanoma**	B16F10 murine melanoma was implanted subcutaneously in C57BL/6 mice (n = 15)	Melittin (*Apis melifera*)	8.5 mg/Kg, 4 injections every other day starting at day 5 (intravenous, Melittin is loaded on molecularly targeted nanoparticles.) S: saline only N: nanoparticle only	Tumor weight was significantly decreased on day 14 compared with S (~88% reduction) and N (~87% reduction) (*p* < 0.01). Decrease in the number of blood vessels in proliferating cells, and significant areas of necrosis in melittin-treated-tumor.	Melittin-loaded nanoparticles cause apoptosis of cancer cell via release of cytochrome c from mitochondria.	[61]
**Wound (Diabetic wound)**	Diabetic 12-week male Balb/c mice wounded on back (n = 45)	BV (*Apis melifera*)	200 µg/kg for 15 day (subcutaneous, on wound area) NC: wound on non-diabetic mice PC: diabetic mice without BV treatment	Degree of wound closure was similar to NC, markedly higher than PC (*p* < 0.05).	Type I collagen expression was significantly recovered in BV-treated diabetic mice compared with PC (*p* < 0.05), lower than NC. Ang-1, Nrf2, p-Tyr, p-eNOS, p-AKT, p-ERK, CD31, CCL2, CCL3, CXCL2 and β-Defensin-2 expression were significantly recovered in BV-treated diabetic mice compared with PC (*p* < 0.05).	[82]
**Wound**	7-week male HR-1 mice wounded on back (n = 30)	BV (*Apis melifera*)	1 µg/gauze (Wound was covered with an equal size of gauze treated with BV for 7 day) NC: untreatedPC: treated with Vaseline	Dramatic decrease of wound size was observed in BV group compared to NC and PC (*p* < 0.05).	Type 1 collagen was remarkably elevated in BV group in comparison to NC and Vaseline. TGF-b1 and fibronectin were significantly decreased in BV group in comparison to control and Vaseline. VEGF was remarkably declined in BV and PC compared to NC (*p* < 0.05).	[79]

Abbreviations: AP-1: activator protein-1, CONC: concentration, DEX: dexamethasone, DFE: *Dermatophagoides farinae* extract, DNCB: 1-chloro-2,4-dinitrobenzene, i.p.: intraperitoneally, i.v.: intravenous, KGF: keratinocyte growth factor, MAPKs: mitogen-activated protein kinases, NC: normal control, OVA: ovalbumin, P. acnes: Propionibacterium acnes, PC: positive control, PLA2: phospholipase A2, s.c:. subcutaneous, TGF-b1: transforming growth factor-b1, TNF-α: tumor necrosis factor- α, Tregs: regulatory T cell, TSLP: thymic stromal lymphopoietin, VEGF: vascular endothelial growth factor.

**Table 3:** In vitro studies on the therapeutic application of bee venom for skin disease.

Disease	Model	Venom/Compound/(Bee Species)	Dose	Results	Mechanism/Molecular Response	Reference
**Acne**	THP-1 cell dealt with heat-killed P. acnes	BV (*Apis melifera*)	Three CONC: 0.1 µg/mL, 1 µg/mL, 5 µg/mL for 48 h	Significant reduction of TNF-α, IL-8 in a concentration-dependent manner (*p* < 0.05). Lowest TNF-α at 5 µg/mL Lowest IL-8 at 1 µg/mL	Not reported	[32]
**Acne**	THP-1 cell dealt with heat-killed P. acnes	BV (*Apis melifera*)	Three CONC: 1 ng/mL, 10 ng/mL, 100 ng/mL for 8 h	Significant reduction of TNF-α, IL-8, IFN-γ at all doses compared to control (*p* < 0.05). Reduced in dose dependent manner.	TLR2 expression significantly suppressed	[107]
**Acne**	THP-1 cell treated with heat-killed P. acnes	Melittin (*Apis melifera*)	Three CONC: 0.1 ng/mL, 0.5 ng/mL, 1 ng/mL. for 8 h	Significant reduction of TNF-α, IL-8 at all doses compared to control (*p* < 0.05). Reduced in dose-dependent manner.	Melittin significantly reduced the phosphorylation of IKK, IκB and NF- κB. Inhibiting the NF- κB signaling pathways.	[107]
**Acne**	HaCat cell treated with heat-killed P. acnes	BV (*Apis melifera*)	Three CONC: 1 ng/mL, 10 ng/mL, 100 ng/mL for 8 h	Significant reduction of TNF-α, IL-8, IFN-γ at 10, 100 ng/mL in comparison to control (*p* < 0.05). Reduced in dose-dependent manner.	TLR2 expression significantly suppressed	[107]
**Acne**	HaCat cell dealt with heat-killed P. acnes	Melittin (*Apis melifera*)	1 µg/mL	Significant reduction of TNF-α, IL-1β, IL-8, IFN-γ compared with control (*p* < 0.05).	TLR2 and 4 expression significantly decreased. Melittin significantly reduced the phosphorylation of IKK, IκB, NF- κB and p-38. Inhibiting the NF-κB and MAPK signaling pathways.	[31]
**Alopecia**	hDPC treated with 0.1% dexamethasone	BV (*Apis melifera*)	Three CONC: 100 ng/mL, 200 ng/mL, 500 ng/mL for 24 h	Significant increase of FGF-2, FGF-7, IGF-1R and VEGF compared with DEX only. (*p* < 0.001–*p* < 0.05). Protein-level of VEGF is increased 1.95-, 2.95-, 2.08 and 1.47-fold with 100, 200, 500 ng/mL BV and 2% minoxidil respectively.	Not reported	[35]
**Atopic dermatitis**	Hacat cell treated with TNF-α and IFN-γ	Melittin (*Apis melifera*)	Three CONC: 0.1 µg/mL, 0.5 µg/mL, 1 µg/mL.	IL-1β, IL-6 and IFN-γ were decreased in a dose-dependent manner. mRNA of CCL17 and CCL22 were significantly decreased in a dose-dependent manner in melitin 0.5 and 1 in comparison to control (*p* < 0.05). pJAK2, pSTAT1 and pSTAT3 expression was decreased in melittin 1 µg/mL	NF- κB DNA-binding activity was markedly reduced.	[54]
**Atopic dermatitis**	Hacat cell treated by 50 ng/mL of IL-4 and IL-13	Melittin (*Apis melifera*)	Three CONC: 0.1 µg/mL, 0.5 µg/mL, 1 µg/mL. for 24 h	Filaggrin expression was remarkably elevated in a dose-dependent manner in all doses compared to control (*p* < 0.05) pSTAT3 expression was significantly decreased in melittin 1 µg/mL	Not reported	[55]
**Melanoma**	Human melanoma A2058 cells	BV (*Apis melifera*)	4 µg/mL	Application of 4 mg/mL BV for 2 h resulted in the death of approximately 80% of A2058 cells.	BV generated reactive oxygen species (ROS) and altered mitochondrial membrane potential transition. BV causes apoptosis in AIF/EndoG-dependent but caspase-independent manner. BV interfered with AKT and MAPK family kinase activation. BV treatment significantly reduced phosphorylated AKT and p38 BV made ER and extracellular Ca^2+^ drift to the cytosol.	[60]
**Photoaging**	HDF cell irradiated by UVB (312 nm)	PLA2-free BV(PBV) and BV (*Apis melifera*)	PBV: 1.5 µg/mL, 3.0 µg/mL, BV 1.5 µg/mL, 3.0 µg/mL	Both PBV and BV significantly restored Type 1 procollagen synthesis in UVB-irradiated HDF cells except for BV 3 μg/mL (*p* < 0.05). Type 1 collagen significantly increased in both BV, PBV compared with control (*p* < 0.05). (Degree: 3.0 BV > 1.5 BV > 3.0 PBV > 1.5 PBV)	PBV and BV treatments significantly attenuated the MMP-1, 2 and 3 expressions (*p* < 0.05). Both PBV and BV significantly inhibited the UVB-stimulated phosphorylations of ERK1/2 and p38 (*p* < 0.05).	[68]
**Photoaging**	Hacat cell irradiated by UVB (312 nm)	PLA2-free BV(PBV) and BV (*Apis melifera*)	PBV: 1.5 µg/mL, 3.0 µg/mL, BV: 1.5 µg/mL, 3.0 µg/mL.	PBV and BV treatments significantly attenuated the MMP-1, 13 expressions (*p* < 0.05). Both PBV and BV significantly inhibited the UVB-stimulated phosphorylations of ERK1/2 and p38 (*p* < 0.05).		[68]
**Photoaging**	HDF cell irradiated by UVB (280–350 nm)	BV (*Apis melifera*)	Three CONC: 0.01 µg/mL, 0.1 µg/mL, 1 µg/mL for 24 h	BV significantly decreased MMP-1 expressions by 50–80% while MMP-3 expression by 50–85% compared to controls (*p* < 0.05). The biggest MMP-1 and MMP-3 inhibitions were observed at a 0.1 µg/mL.	Not reported	[67]
**Vitiligo**	Human epidermal melanocyte	BV (*Apis melifera*)	10 µg/mL	Melanocyte proliferation and melanin content were remarkably increased compared to control (*p* < 0.05), similar to melanocyte treated with 10 µM forskolin but no more than.	Forskolin increased the cAMP level 40-fold, but BV only tripled. Based on this, the cAMP level does not appear to be the deciding factor	[89]

Abbreviations: AIF: apoptosis-inducing factor, AKT: protein kinase B, cAMP: cyclic adenosine monophosphate, CONC: concentration, DEX: dexamethasone, EndoG: endonuclease G, ER: endoplasmic reticulum, ERK1/2: extracellular signal-regulated kinase 1 and 2, FGF: fibroblast growth factor, HaCat: human keratocyte, HDF: human dermal fibroblasts, hDPC: human dermal papilla cell, HEK: human epidermal keratinocyte, IGF-1R: insulin-like growth factor 1 receptor, MAPK: mitogen-activated protein kinase, *P. acnes*: *Propionibacterium acnes*, pJAK: phosphorylated janus kinases, pSTAT3: phosphorylated signal transducer and activator of transcription, TLR2: Toll-like receptor 2, UVB: ultraviolet, VEGF: vascular endothelial growth factor.

**Table 4:** Adverse effects reported in clinical studies and in in vivo studies.

Disease	Type of Study	Venom/Compound/(Bee Species)	Adverse Effect (Severity)	Reference
**Atopic dermatitis**	Clinical	Emollient containing BV (*Apis melifera*)	Irritation, pruritus, erythema, urticaria and disease exacerbation (mild). No significant differences in the incidence compared with control.	[49]
**Psoriasis**	Clinical	BV (*Apis melifera*)	Mild pain, redness and swelling at the site of apitheraphy injection	[75]
**Psoriasis**	Clinical	BV (*Apis melifera*)	4patients experienced itching but not significant. No systemic adverse effect.	[71]
**Scleroderma**	Clinical	BV (*Apis melifera*)	Slight itchiness at the location of inoculation for 1 half-day.	[64]
